# Raising positive expectations helps patients with minor ailments: A cross-sectional study

**DOI:** 10.1186/1471-2296-9-38

**Published:** 2008-06-30

**Authors:** Thijs Fassaert, Sandra van Dulmen, François Schellevis, Liesbeth van der Jagt, Jozien Bensing

**Affiliations:** 1 NIVEL (Netherlands Institute for Health Services Research), Utrecht, The Netherlands; 2 Department of General Practice, VU University Medical Centre, Amsterdam, The Netherlands; 3 NHG (Dutch College of General Practitioners), Utrecht, The Netherlands; 4 Department of Health Psychology, Utrecht University, Utrecht, The Netherlands

## Abstract

**Background:**

Consultations for minor ailments constitute a large part of the workload of general practitioners (GPs). As medical interventions are not always available, specific communication strategies, such as active listening and positive communication, might help GPs to handle these problems adequately. This study examines to what extent GPs display both strategies during consultations for minor ailments and investigates how each of these relate to the patients' perceived health, consultation frequency and medication adherence.

**Methods:**

524 videotaped consultations between Dutch GPs and patients aged 18 years or older were selected. All patients presented a minor ailment, and none of them suffered from a diagnosed chronic illness. The observation protocol included the validated Active Listening Observation Scale (ALOS-global), as well as three domains of positive communication, i.e. providing reassurance, a clear explanation, and a favourable prognosis. Patients completed several questionnaires before, immediately after, and two weeks after the consultation. These included measures for state anxiety (STAI), functional health status (COOP/WONCA charts) and medication adherence (MAQ). Consultation frequency was available from an ongoing patient registration. Data were analysed using multivariate regression analyses.

**Results:**

Reassurance was related to patients' better overall health. Providing a favourable prognosis was linked to patients feeling better, but only when accompanied by a clear explanation of the complaints. A clear explanation was also related to patients feeling better and less anxious, except when patients reported a low mood pre-visit. Active listening alone was positively associated with patients feeling worse. Among patients in a good mood state, active listening was associated with less adherence.

**Conclusion:**

To some extent, it seems helpful when GPs are at the same time clear *and *optimistic about the nature and course of minor ailments. Yet, it does not seem helpful always and in all cases, e.g. when patients feel low upon entering the consulting room. Although communication strategies might to some extent contribute to the management of minor ailments, the results of this observational study also indicate that it is important for a physician to pay attention to the mood of the patient who enters the consulting room.

## Background

Patients visit their general practitioner (GP) for minor ailments relatively often [1,2]. Depending on the definition applied, minor ailments can account for up to 40 to 80 percent of all consultations, thereby influencing the workload of GPs significantly [3-6]. In Great Britain, for example, an estimated 100 to 150 million consultations are for conditions that are potentially self-treatable [7] or that can be managed by providing self-care advice. For example, general guidelines for treating acute a-specific low back pain in general practice recommend that, besides offering reassurance, GPs should start to prescribe acetaminophen (paracetamol) and to advise patients to take this at regular intervals and in usual doses [8,9]. Apart from that, GPs are of the opinion that minor ailments do not require much attention [4]. Also, patients consulting their GP with minor illnesses often have no high expectations of GP-care [2]. As a result, visits for minor ailments usually proceed quite unsatisfactory for both GPs and patients [10].

The decision whether or not to seek health care for minor ailments is thought to be the outcome of a complex process, influenced by a wide range of factors [9]. This process itself might be able to influence health outcomes, as can for example be derived from Leventhal et al. [11], who developed the self-regulatory model. They argued that, when confronted with illness, patients respond both with emotions (e.g. worries about the nature of the complaints) and cognitions (e.g. explanations). Indeed, previous research showed that among reasons to consult were patients' anxiety about the severity of the problem and the need for an adequate explanation [12-14]. According to the self-regulatory model, patients eventually develop illness perceptions, typically defined by ideas about (a) illness identity or diagnosis, (b) illness duration, (c), health consequences, (d) possible causes and (e) cure or containment. Persisting questions and worries regarding one of these elements, such as the nature of the problem, can interfere with patients' spontaneous recovery and the natural course of the illness [15,16]. As a matter of fact, anxiety has been directly associated with ill health [17].

Therefore, when confronted with patients presenting minor ailments, GPs should address patients' concerns and questions at an early stage. Following patients' reasons for consulting, a two-way communication strategy seems to be required from the GP, including (i) affective communication to attend to patients' concerns and (ii) instrumental communication for addressing informational needs [18,19]. The present study focuses on active listening as a form of affective communication aimed at recognising and attending to patients' emotional concerns [20], and on positive communication as a form of instrumental communication, by which GPs provide a clear explanation about the cause of an ailment, reassurance about its harmless nature and information about when it will disappear [21,22]. Physician-patient communication has been found before to improve health outcomes [23]. For example, attentive, friendly and empathic practitioners may be considered more effective than those who keep consultations formal [24]. Additionally, a strong association has been found between GPs' reassurance and explanation on the one hand, and patient satisfaction, compliance, comprehension and perception of a good interpersonal relationship on the other [25]. Results of the classical study by Thomas [21] suggest that offering a reassuring statement about the absence of a serious underlying disease, in combination with a firm diagnosis and a definitive and favourable prognosis, shortens the illness duration in symptomatic patients and increases their satisfaction with the visit.

The evidence for the favourable influence of positive communication and active listening on patient outcomes is, however, scarce. Moreover, a recent randomized trial [22] failed to replicate the findings of Thomas' classical study [21]. In addition, previous studies generally did not take into account the emotional state of patients, while there are indications that patients' emotions interact with physicians' communication style [26,27]. The evidence on the effectiveness of being positive and supportive is therefore not conclusive. This observational study will therefore (a) examine how GPs communicate during everyday consultations, initial or follow-up, for minor ailments and (b) examine the relationship between GPs' positive communication/active listening, and patients' perceived health, consultation frequency and medication adherence. Consultations for chronic conditions, in which communication is likely to play a very different role, were excluded. Positive communication and active listening by the GP were expected to be related to less anxiety immediately after the consultation. Additionally, it was expected that these communication strategies would shorten the illness duration and consequently result in less healthcare utilization. Finally, it was expected that active listening and positive communication were associated with better adherence to medication.

## Methods

### Participants

The present study was set within the Second Dutch National Survey of General Practice (DNSGP-2), carried out in 2001 by NIVEL [28,29]. DNSGP-2 was carried out in 104 general practices in the Netherlands, comprising 195 GPs. The selection of practices and GPs was based on region (north, central and south), level of urbanisation and practice type (working alone, group-practice), and was considered to be nationally representative. GPs working alone were somewhat underrepresented [28]. Of all participating GPs, 142 were observed by video camera during consultations. Per GP, 15 to 20 consultations were recorded, resulting in 2784 recordings. Health problems were ICPC-coded [30]. In the end, 27% of the GPs who participated in the DNSGP-2 did not obtain informed consent from their patients to videotape consultations. Although this may have induced bias, for example because some GPs were more interested in communication than others, this is contradicted by the representativity of the participating GPs [31].

For this study, only recordings with patients of 18 years and older and consulting for minor ailments were selected. Minor ailments were defined as frequently presented health problems, without any underlying chronic disease. To meet the first criterion, a consultation was included if a patient's primary self-reported health problem was common, indicated by high incidence and prevalence rates within DNSGP-2. Previous research within the total research population of DNSGP-2 (N = 375,899 patients) showed that complaints in ICPC-chapters D, L, R and S (digestive, musculoskeletal, respiratory and skin problems, respectively) were presented most often in general practice [32]. The second criterion was met when the GP's corresponding diagnosis after the consultation did not indicate a chronic disease. For this purpose, a cluster of chronic diseases (e.g. cancer and diabetes) coded by ICPC was selected [33], and patients with an underlying chronic disease were excluded. These criteria resulted in a selection of 524 videotaped consultations (139 GPs with on the average 3.8 patients each; range 1–8).

### Procedure

The patients whose consultation was recorded completed a questionnaire immediately before the consultation – about sociodemographic characteristics, functional health status during two weeks preceding the consultation, and pre-consultation anxiety. Immediately after the consultation, patients completed a second questionnaire, to evaluate post-consultation anxiety levels. Finally, patients received a third questionnaire two weeks after the consultation to measure functional health status again, and to measure their level of adherence to the medication prescribed during the videotaped visit (if any). All recorded consultations were observed using a checklist measuring positive communication and active listening.

### Communication strategies

#### Positive communication

Comparable to Thomas [21] and Knipschild and Arntz [22], a positive consultation was defined as a consultation in which a GP (a) explicitly excluded the possibility of any serious disease underlying the presented symptoms ('reassurance'), (b) gave a clear explanation about the possible causes of the symptoms ('clear explanation'), and (c) was optimistic and outspoken about the time in which the problem was going to be settled ('favourable prognosis'). Those three domains were measured on 5-point Likert-type scales ranging from 'strongly disagree' to 'strongly agree' (Table 1). Although the domains were originally constructed to form one *index *score of positive communication, Cronbach's alpha for a three-item scale was as low as 0.12. Results of an additional factor analysis, with a forced three-factor solution (results not shown), indicated that each item had factor loadings exceeding 0.50 on separate factors. The domains were therefore entered in the analyses separately. Twenty consultations were randomly selected and viewed by a second rater to assess interobserver reliability (Cohen's Kappa). Each had a Kappa of 0.40 for the dichotomized answering scales ('never', 'rarely' and 'neutral' versus 'regularly' and 'always'), indicating moderate agreement beyond chance [34].

**Table 1:** Items and response rates (%), missing values and means for positive communication and active listening items (N = 524)

	*Totally disagree*	*Disagree*	*Neutral*	*Agree*	*Totally agree*	*Missing*	**Mean (Sd)**
Positive communication **The GP**...							
1. tells the patient that he/she is not suffering from a serious illness	1.5	16.2	48.1	20.0	13.9	0.2	3.29 (.95)
2. communicates the cause of the complaints clearly	7.6	22.7	38.0	27.3	4.0	0.4	2.97 (.99)
3. makes a favourable prognosis on the course of the complaints	.2	6.9	55.9	30.3	6.7	0	3.36 (.72)

	*Never*	*Rarely*	*Sometimes*	*Regularly*	*Always*	*Missing*	**Mean (Sd)**
Active listening **The GP**...							
1. shows not to be distracted during the consultation	1.0	7.1	30.5	37.8	23.3	0.4	3.76 (.92)
2. is not off-hand or hasty	1.1	8.8	33.4	39.3	17.0	0.4	3.62 (.91)
3. listens attentively	1.5	8.4	22.1	38.4	29.6	0	3.86 (.99)
4. gives the patient time and space to present his problem	2.1	13.0	26.1	41.0	17.6	0.2	3.59 (.99)
5. uses exploring questions	6.5	34.0	30.7	23.5	5.0	0.4	3.30 (.82)
6. is good in leading the conversation	2.3	24.8	34.7	31.5	5.5	1.1	3.13 (.93)
7. expresses understanding non-verbally	9.2	26.9	27.7	30.0	6.1	0.2	2.97 (1.09)

#### Active listening

Active listening was measured by the global Active Listening Observation Scale (ALOS-global), a newly developed and validated observation scale, for which active listening was defined as a GP's 'attentiveness to and acknowledgement of the patient's suffering' [20]. The ALOS-global consists of seven 5-point Likert-scaled items (extremes labelled 'never' and 'always'). Internal consistency for these items was good (Cronbach's alpha 0.84). Based on the same random sample of twenty videos, inter-observer agreement was moderately good beyond chance (average Kappa = 0.53, range 0.43–0.62). Items are depicted in Table 1.

### Outcome measures

#### Functional Health

The COOP/WONCA charts measure six core aspects of functional health status, developed for application in primary care [35,36]. For each chart, patients are asked to rate their status on an ordinal 5-point scale, from 1 (no limitation at all) to 5 (severely limited). Previous studies have established good psychometric properties for the COOP/WONCA charts [33,37,38], which can be used for both scale analysis, as well as item-by-item analysis [39,40]. During DNSGP-2, all charts were administered. For the purpose of this study, the items *mood*, *physical health *and *overall health *were selected. As a higher score indicates more limitations, these charts will be referred to as *low *mood, *ill *physical health and *ill *overall health.

#### State Anxiety

State anxiety was measured using the shortened Dutch version of the State-Trait Anxiety Inventory (STAI) [41]. The full English questionnaire was developed by Spielberger [42], the shortened English version by Marteau and Bekker [43], the validated Dutch version by van der Ploeg et al. [41], and the shortened Dutch versions by Knippenberg et al. [44] and van der Bij et al. [45]. State anxiety was measured by 10 items, rated on 4-point answering scales (anchored 'not at all' to 'very much so'). Items ask respondents to state how they currently feel regarding 10 statements that are worded either positively (e.g. 'I feel calm') or negatively (e.g. 'I feel strained'). Positively phrased items were recoded, and sum scores were calculated (range 10 – 40). Cronbach's alpha's were 0.90 and 0.89, before and immediately after the consultation, respectively.

#### Medication adherence and consultation rates

Medication adherence was measured using the Medication Adherence Questionnaire (MAQ) [46]. The MAQ is a reliable and valid self-report questionnaire which measures whether patients (i) forgot to take their medication, (ii) were careless in taking them, (iii) stopped drug-intake when they felt better or (iv) stopped medication when they felt worse. Non-adherence was defined as a positive answer on at least one of these items. Three hundred thirty-five patients indicated they had received a prescription for medication during the videotaped consultation, of whom 318 reported complete information on all determinants.

Consultation rates were obtained by linking the video-recorded consultations to the specific illness-episode in the electronic medical records (EMR) of GPs [28]. Data from these EMRs are continuously being collected on behalf of the Dutch National Information Network of General Practice (LINH) [47]. Illness episodes in LINH are defined as all encounters connected to the management of a specific health problem, in this case the minor ailment. Thus, each videotaped consultation was uniquely connected to a specific number of consultations. For example, if a patient consulted for back pain, and returned three times for a check-up, there was one illness-episode (for back pain), consisting of four consultations, of which either one was recorded. By linking the video database to the EMRs, information was available for the total number of consultations within the same illness-episode (contact frequency) and for whether or not the patient returned to the GP for the same condition after the recorded consultation (follow-up consultation). Complete data records with respect to both variables was available for 409 and 250 patients, respectively.

### Statistical analysis

Multiple linear regression analyses were performed to determine whether positive communication and active listening were associated with less anxiety and better functional health status. Multiple logistic regression analyses were performed to determine the relationship between communication, medication adherence and consultation rates. Each regression analysis included, if applicable, pre-visit values of the outcome measures. Additionally, patient-characteristics (age, gender (1 = male) and education level (low-middle-high)) and pre-visit overall health status (COOP/WONCA) were added, as these could act as possible confounders. Communication variable(s) were included as main effects and as interaction-terms with pre-consultation mood of patients, as the association between communication and outcome measures was expected to depend on patients' pre-consultation mood state. Finally, we examined the interaction between positive communication variables, because the results by Thomas' study seem to suggest that especially the combination of (a) offering reassurance, (b) a firm diagnosis and (c) a favourable prognosis shortens the illness duration, rather than any of these dimensions alone. Analyses were performed using SPSS version 11.5. Only significant results were presented, as well as non-significant main effects if they were involved in a significant interaction effect. Correction for clustering (multilevel analysis) was considered, but intra-class correlations were too low (0.04 on average, in a range from 0.00 to 0.10) and the number of consultations per GP was too small (139 GPs with on the average 3.8 patients each; range 1–8). Kreft [48] argues that 150 groups with 5 observations within each group should be sufficient to obtain a power of 0.90, but fewer observations (either groups or individuals) show a rapid decline of power for the detection of cross-level interactions [49].

#### Non-response analysis

Patients who did not complete the questionnaire before the consultation were significantly older (51.2 ± 17.7) than patients who did (44.8 ± 16.1, p < 0.01). No differences were found with respect to patients' gender and education. Patients whose information from immediately after the consultation was missing were again significantly older (52.7 ± 18.5 compared to responders (45.1 ± 16.1, p < 0.01), and more likely to be female (Chi^2 ^= 4.07, p < 0.05). There were no differences concerning education. With respect to the third questionnaire, sent two weeks after the consultation, there was a non-response of approximately 32.2% of the original study sample. Analysis revealed that this time non-responders were significantly younger (40.7 years ± 15.2) compared to responders (48.5 years ± 16.6). There were no differences concerning patients' gender and education.

## Results

The selected consultations included 139 GPs, of whom 105 were male (75.5%). Male GPs were on average 47.9 years old (± 5.7), female GPs were younger (44.1 years ± 7.0) (p < 0.01). About half the GPs worked fulltime (51.1%). Most GPs (38.8%) worked in a group-practice, while 31.7% and 29.5% of the GPs worked solo or with one partner, respectively. GPs were settled for at least one year and at most 32 years (average 15.6 years ± 8.3).

Two-hundred ninety-nine patients whose consultations were recorded (57.1%) were female (Table 2). Mean age at the time of the consultation was 45.7 years (± 16.6) for women and 46.5 years for men (± 16.6) (n.s.). Sixty-three patients (12.0%) presented a minor ailment related to the digestive system, while 270 (51.5%), 130 (22.8%) and 61 (11.6%) minor ailments were related to the musculoskeletal system, the respiratory system and the skin, respectively. Seventeen percent of the patients who received a prescription for medication during the videotaped consultation reported a type of medication non-adherence. From the available registration data it could be derived that almost twenty-four percent of the patients had more than one contact within the same illness-episode. Additionally, one-third of the consultations was followed up by another contact. Table 3 shows that before the consultation patients rated their mood state best, followed by their physical and overall health. The same applies to the health state after the consultation.

**Table 2:** Patient-characteristics compared to total study population (1)

	Sample		DNSGP-2 video-study	
		
	N	%	N	%
**Sex**	524		2784	
Male		42.9		41.2
Female		57.1		58.8
**Age**	524		2784	
18–44		50.8		42.9
45–64		31.8		35.5
65 and older		17.4		21.6
**Education**	524		2784	
Low		21.5		28.3
Middle		64.0		56.6
High		14.6		15.1
**ICPC-chapter**	524		2784	
Digestive		12.0		5.9
Musculoskeletal		51.5		21.9
Respiratory		22.8		13.6
Skin		11.7		11.1
Other		-		47.5
*Medication adherence*	335		2007	
adherence		83.0		86.8
non-adherence		17.0		13.2
**Contact frequency**	435		2225	
Once		76.1		74.2
More than once		23.9		25.8
**Follow-up**	263		831	
No follow-up		66.9		74.2
Follow-up		33.1		25.8

**Table 3:** Patient-characteristics compared to total study population (2)

	Sample		DNSGP-2 video-study	
		
	N	Mean (sd.)	N	Mean (sd.)
**Health status (pre-visit)**				
Mood	503	2.1 (1.2)	2184	2.2 (1.2)
Physical health	502	2.5 (1.3)	2167	2.6 (1.3)
Overall health	506	3.1 (1.1)	2203	3.2 (1.1)
**Health status (post-visit)**				
Mood	349	2.0 (1.1)	1593	2.0 (1.1)
Physical health	347	2.6 (1.2)	1576	2.6 (1.1)
Overall health	348	3.2 (1.0)	1591	3.1 (1.0)
**State Anxiety (pre-visit)**	427	18.7 (5.6)	1781	19.2 (5.2)
**State Anxiety (post-visit)**	460	18.2 (5.8)	1990	18.3 (6.0)

### Positive communication and active listening

Responses with respect to communication are depicted in Table 1. The results indicate that during one-third of the consultations, the GPs stated that the patient did not suffer from any serious disease and that they believed the patient would be better soon. In almost half the visits the GPs seemed to score neither positive nor negative on these skills. Also during one third of the consultations, the GPs communicated the cause of the complaints clearly. Yet, in the same number of visits, the GPs' performance was rated negatively on this point. In almost two-thirds of the consultations, the GPs communicated a clear explanation for the cause of the presented health problem. In more than half of the cases, the GPs showed not to be distracted or off-hand, they listened actively and gave the patients space to present the problem. Less often, the GPs asked exploring questions or communicated their understanding of the patients' feelings nonverbally.

### Communication, health and healthcare utilization

In general, regression analyses showed that the pre-consultation values of the outcome measures were strongly related to the post-consultation values (Table 4). Additionally, feeling unhealthy before the consultation had an important negative influence on several outcome measures. Worse overall health was related to more post-consultation anxiety, worse mood and worse physical health. Regression analyses also showed that a higher age was related to an increased chance of follow-up contacts. Male gender was associated with better overall health.

**Table 4:** Associations of sociodemographic and communication variables with post-visit measures of health

Dependent variable	Independent variable	β	Std. Error	p
Anxiety	Anxiety (pre-visit)	.62	.04	.00
	Ill overall health (pre-visit)	.48	.21	.02
	Low mood (pre-visit)	.54	.22	.01
	Clear explanation	-.09	.21	n.s.
	Clear explanation × Low mood (pre-visit)	.55	.23	.02
Low mood	Low mood (pre-visit)	.45	.05	.00
	Age	-.01	.00	.03
	Ill overall health (pre-visit)	.17	.05	.00
	Clear explanation	-.02	.05	n.s.
	Favourable prognosis	-.11	.07	n.s.
	Clear explanation × Favourable prognosis	-.12	.05	.02
Ill physical health	Ill physical health (pre-visit)	.48	.05	.00
	Ill overall health (pre-visit)	.13	.05	.01
	Active listening	.03	.01	.02
	Clear explanation	-.05	.05	n.s.
	Favourable prognosis	.08	.07	n.s.
	Clear explanation × Favourable prognosis	-.12	.05	.02
Ill overall health	Ill overall health (pre-visit)	.60	.04	.00
	Low mood (pre-visit)	.01	.04	n.s.
	Gender (0 = male, 1= female)	-.25	.09	.01
	Active listening	.03	.01	.00
	Reassurance	-.12	.05	.01
	Clear explanation	-.12	.05	.01
	Favourable prognosis	-.02	.06	n.s.
	Clear explanation × Favourable prognosis	-.11	.04	.02
	Clear explanation × Low mood (pre-visit)	.10	.05	.03

n.s. = not significant

The results furthermore showed that the GPs' reassurance was related with better overall health (Table 4). Additionally, there was interaction between the GP providing a 'clear explanation' and him or her giving a 'favourable prognosis' (Table 4). Secondary analyses of these interaction effects indicated that GPs' clear explanation was only beneficial – in terms of associations with better mood, better physical health, and better overall health of patients – when GPs also provided a positive prognosis. Furthermore, interaction effects were found between GPs' clear explanations and patients' mood before the consultation. Giving a clear explanation was positively associated with post-consultation anxiety and ill overall health among patients who felt low previous to the consultation. In this subgroup of patients who felt low, a favourable prognosis was also associated with the occurrence of more than one contact for the same complaint (Table 5). Finally, active listening was associated with feeling worse, both physically and overall. No other main effects of active listening were found. However, there was a significant interaction between active listening and mood regarding medication adherence; among patients in a good mood state, the GP's active listening was associated with less adherence (Table 5). Among patients feeling low, no association between active listening and adherence was found.

**Table 5:** Associations of sociodemographic and communication variables with adherence and consultation rate

Dependent variable	Predictor	β	SE β	Wald's χ^2^	df	P	OR
**Medication adherence**	Ill overall health	-0.36	0.16	4.90	1	0.03	0.70
0 = adherence	Low mood	0.33	0.15	5.06	1	0.03	1.39
1 = non-adherence	Active listening	0.03	0.04	0.58	1	n.s.	1.03
	Active listening × Low mood	-0.39	0.16	6.21	1	0.01	0.68

**Contact frequency**	Low mood	.05	.12	.16	1	n.s.	n.a.
0 = once	Favourable prognosis	.05	.19	.08	1	n.s.	n.a.
1 = more than once	Favourable prognosis × Low mood	.43	.15	7.82	1	.01	1.53

**Follow-up contact**	Age	.03	.01	8.72	1	.00	1.03
0 = no follow-up							
1 = follow-up contact							

n.s. = not significant

n.a. = not applicable

## Discussion

In this study, we examined the extent to which GPs made use of positive communication and active listening during consultations in which patients presented (non-chronic) minor ailments, and the relation between communication and patient outcomes. To our knowledge, only few studies have examined these relationships in real-life medical practice and in this specific population. Patients with minor ailments are often perceived as difficult to cope with by GPs, and determine their daily workload to a considerable extent. Because it is the interaction between the doctor and the patient, and not just the patient himself, that determines whether an encounter is perceived as 'difficult' [50], we hypothesized that communication directed at improving patients' medication adherence might help GPs to cope with the workload resulting from minor ailments. For this study, positive communication and active listening were expected to show positive effects on the health of, and healthcare utilization by, patients with minor ailments.

The results indicated that, to some extent, domains of positive communication (i.e. reassurance, a clear explanation and a favourable prognosis) were associated with preferable patient outcomes. The provision of reassurance appeared to contribute to patients feeling better; a clear explanation and a favourable prognosis appeared to alleviate patients' functional health status as well. In a recent review, similar effects of the interaction between different communication skills were found [24].

Yet, contrary to expectations, active listening seemed to be negatively associated with self-reported health. Moreover, active listening was associated with non-adherence to medication if patients felt good prior to the consultation. These results suggest that active listening should not be a GP's first reaction to patients consulting their GP with minor ailments. One possibility is that some patients may interpret active listening by a GP as necessary concern on his or her part [27], suggesting a serious nature of the complaints and thus eliciting negative feelings in patients in whom such feelings are initially absent. Confirming these believes in other, more serious conditions might be correct or even functional, but probably not for self-limiting non-chronic conditions that usually lack the need for a medical intervention. Instead, telling the patient what is going on and when complaints are expected to disappear seem to have more impact.

In addition, some of the results suggested that patients who experience negative emotions before the consultation should be treated differently from patients who do not experience such feelings. This arose from the fact that we were able to take into account patients' mood before the consultation, which is an advantage upon previous studies which often failed to take into account the impact of patients' emotional state on the effects of communication [26,27]. The interaction between patients' pre-consultation mood and positive communication suggests that positive communication may be effective, but that it should not be promoted unconditionally. Rather, this finding indicates how important it is for a GP to be sensitive to the emotional state of a patient.

Given these restrictions, we found no conclusive evidence of a direct relation between positive communication and problem duration, in contrast to Thomas [21], but in accordance with Knipschild and Arntz [22]. In fact, ill physical health two weeks after the consultation was best predicted by patients' functional health status prior to the consultation. The chance of occurrence of a follow-up consultation for the same problem was best predicted by patients' age.

There are, however, some differences between our study and the studies mentioned above which should be taken into account. For example, the patient and GP populations in our study were more heterogeneous compared to the other studies. Thomas [21] studied 200 patients and only one physician, namely himself, while Knipschild and Arntz [22] included 17 doctors and 128 patients. Additionally, Thomas included all symptomatic patients in whom no definite diagnosis could be made, while Knipschild and Arntz restricted their population to four symptomatic diagnoses: headache, sore throat, abdominal pain and pain related to movement [21,22]. Furthermore, we selected somewhat different outcome measures compared with the other studies. For example, we measured self-reported functional health, which is not the same as specifically checking with a patient whether a particular minor ailment has resolved after two weeks. On the other hand, we used outcome measures, such as healthcare utilization, that could be linked specifically to the minor ailment in question. Finally, our study was cross-sectional, while the other studies were randomised trials, which is a prerequisite for establishing causal relationships.

This study has some limitations as well. First, the items that were used to measure positive communication have not been used before. We therefore encourage efforts to further validate them. Second, due to time constraints the analyses for interrater reliability were restricted to twenty consultations. We acknowledge that it would have been better if more observations were done to establish interrater agreement. In addition to this point, interrater agreement was moderately high, suggesting room for improvement regarding the objectivity of the observations. However, the main observer for this study was always blind with respect to the selected outcome measures, which suggests that occurrence of systematic bias is unlikely. Third, the cross-sectional design complicated the interpretation of the results in terms of causality. For example, positive communication by the GP (e.g. providing a favourable prognosis) may have been induced by the minor character of the complaint, without necessarily affecting the outcome, because minor ailments have a natural tendency to cure spontaneously [1]. In other words, the minor ailment might have resolved anyway, regardless of the communication style of the GP. Likewise, instead of resulting in worse self-reported health, active listening by the GP may have actually been triggered by patients' worse health condition. Fourth, we may have missed minor ailments by excluding all patients with a chronic condition who presented a minor ailment that was not related to their chronic illness. In that respect, one might argue that patients with chronic health conditions actually do belong to our sample. Finally, interpretation of the results is also complicated by the non-response during subsequent measurements of anxiety, health status and adherence to treatment. Especially the questionnaire two weeks after the consultation resulted in a high non-response. As a consequence, the data with respect to adherence to medication might have been subjected to selection bias, resulting in an overestimation of the level of adherence. The results should therefore be interpreted cautiously, and we encourage more research in this field in order to determine the validity of our findings.

## Conclusion

For most patients with minor ailments, it seems generally helpful when a GP is clear *and *optimistic about the nature and course of the complaints at the same time. Yet, it does not seem helpful in all cases, all the time. The finding that negative emotions play an important role in these relations, suggests that patients presenting minor ailments should not automatically be responded to with positive communication, nor with active listening. Patients without negative emotions before the consultation seem to benefit from a more positive consultation style of the GP. Patients who feel low upon entering the consulting room appear less affected by positive communication. More effort is needed to determine the reproducibility of these results, and to determine to what extent they are relevant for clinical practice.

## Competing interests

The authors declare that they have no competing interests.

## Authors' contributions

The study was designed by SvD. TF conducted the study, performed the analysis and then drafted the manuscript together with SvD. FS, LvdJ and JB contributed to the manuscript with their interpretation of results and comments on earlier drafts. All authors read and approved the final manuscript.

## Pre-publication history

The pre-publication history for this paper can be accessed here:


